# Prevalence and Characteristics of Breathing Pattern Disorders in Adults With Refractory Chronic Cough or Inducible Laryngeal Obstruction: A Systematic Review

**DOI:** 10.1111/1460-6984.70365

**Published:** 2026-11-11

**Authors:** Kirsty Bui, Hannah Harvey, Jennifer Butler, Jemma Haines, Gemma Clunie

**Affiliations:** ^1^ Frimley Health NHS Foundation Trust Slough UK; ^2^ Buckinghamshire New University Uxbridge UK; ^3^ Birmingham City University Birmingham UK; ^4^ Northumbria Healthcare NHS Foundation Trust North Tyneside UK; ^5^ Manchester University NHS Foundation Trust Manchester UK; ^6^ Imperial College Healthcare NHS Trust and Imperial College London London UK

**Keywords:** Breathing pattern disorder, chronic cough, inducible laryngeal obstruction, exercise‐induced laryngeal obstruction, upper airway disorders, systematic review

## Abstract

**Background:**

Breathing Pattern Disorders (BPD) have been reported in association with upper airway disorders, including Refractory Chronic Cough (RCC) and Inducible Laryngeal Obstruction (ILO). However, the prevalence and clinical characteristics of BPD reported within these populations is unclear, limiting understanding of its occurrence, presentation, and implications for clinical assessment and management.

**Aim:**

To answer the following research question—‘What is the reported prevalence and characteristics of breathing pattern disorders in adults with RCC or ILO?'

**Methods:**

The protocol was registered with PROSPERO: CRD420261419679 and reported in accordance with PRISMA guidelines. Five bibliographic databases (MEDLINE, CINAHL, PsycINFO, Cochrane Library, and WoS) were systematically searched to identify studies published from 2016 to 2026. All titles and abstracts were screened by one reviewer, with 10% screened by two additional reviewers. Studies reporting the prevalence and/or clinical characteristics of BPD in adults with RCC or ILO were eligible. Following de‐duplication, 151 records were identified, with four meeting the eligibility criteria. Methodological quality was assessed using the Joanna Briggs Institute Critical Appraisal Checklist for Studies Reporting Prevalence Data, and certainty of evidence was assessed using the Grading of Recommendations Assessment, Development, and Evaluations (GRADE) approach.

**Main Contribution:**

BPD was reported to be a common and clinically relevant comorbidity in RCC and ILO cohorts, with 73% of RCC patients and 69%–89% of ILO patients demonstrating at least one breathing abnormality. Across studies, abnormalities in breathing region (thoracic vs abdominal) and route (oral vs nasal) were consistently identified as prominent features in altered breathing patterns. Similar breathing pattern characteristics were observed across RCC and ILO cohorts, suggesting overlapping physiological features. However, interpretation is limited by the small evidence base and lack of standardised assessment.

**Conclusion:**

BPD appears to be a frequently identified comorbidity in RCC and ILO; however, interpretation is limited by the small evidence base and lack of standardised assessment approaches. Despite this, the included studies identified clinically relevant BPD characteristics and suggest potential associations between abnormal breathing patterns and laryngeal dysfunction, influenced by contributing factors. Diagnostic overlap between BPD, RCC, and ILO may complicate assessment and management, particularly in centres without access to laryngoscopy. Clinicians should consider comprehensive breathing assessment within routine upper airway assessment.

**WHAT THIS PAPER ADDS:**

*What is already known on this subject*

- Breathing Pattern Disorders (BPD) are associated with upper airway disorders, including Refractory Chronic Cough (RCC) and Inducible Laryngeal Obstruction (ILO). However, the prevalence and clinical characteristics of BPD reported within these populations is unclear.

*What this paper adds to existing knowledge*

- This study systematically reviewed the reported prevalence and characteristics of BPD in adults with RCC and ILO. The findings indicate that BPD is commonly reported in these populations, with overlapping clinical features observed across conditions.

*What are the potential or actual clinical implications of this work?*

- These findings emphasise the need for standardised assessment protocols to support accurate clinical differentiation, particularly in centres that do not have access to laryngoscopy for definitive diagnosis. Clinicians should be aware of the potential for crossover between BPD, RCC, and ILO and consider comprehensive breathing assessment as part of routine upper airway assessment and management.

## 1 Introduction

Upper airway disorders encompass a group of conditions characterised by laryngeal dysfunction, often associated with altered laryngeal sensation and airway control (Hull et al. 2016). This review focuses on Refractory Chronic Cough (RCC), defined as a cough lasting longer than eight weeks which persists despite addressing comorbidities or where no comorbidities are identified (Parker et al. 2023) and Inducible Laryngeal Obstruction (ILO), characterised by transient, reversible laryngeal narrowing occurring in a compatible clinical context (Leong et al. 2023). These conditions can co‐exist with other conditions or present alone and are not only a ‘symptom’ of other diseases (Lee et al. 2020). Comorbidities include voice changes, swallowing difficulties (Hull et al. 2016), other upper and/or lower airway disorders (Halvorsen et al. 2017), but also abnormal breathing patterns (Selby et al. 2019).

Abnormal breathing patterns relate to an alteration in the normal biomechanical pattern of breathing (Barker and Everard 2015). This includes elevated respiratory rate/hyperventilation (Todd et al. 2018), a thoracic‐dominant breathing pattern, accessory muscle usage (Denton et al. 2019; Lee et al. 2020) and mouth breathing (Hallani et al. 2008). However, lack of terminology consensus between clinicians and the literature impacts on research comparability. In their 2025 survey of clinicians and patients, Grillo et al. found that ‘breathing pattern disorder’ was the most widely recognised term between clinicians, followed by ‘dysfunctional breathing’. Other terms that have been used include hyperventilation syndrome, abnormal breathing, or respiratory pattern disorder; however, this list is not exhaustive. For this review the term Breathing Pattern Disorder (BPD) will be used throughout for simplicity to represent all the abnormal breathing patterns defined above.

Sufficient breath is critical for the proper execution of vocal and laryngeal tasks and Speech and Language Therapists (SLTs) routinely address breathing strategies as part of their therapeutic interventions. Clinical observation suggests that BPD can co‐occur with upper airway disorders. Despite clinical recognition of this overlap, the prevalence and specific characteristics of BPD across RCC and ILO populations have not previously been systematically synthesised. For these reasons, a systematic review was undertaken, and the following research question was formulated: ‘What are the reported prevalence and characteristics of BPD in adults with RCC or ILO?'

## 2 Methods

A systematic literature search was conducted in on the 16th of April 2026 by one of the review team in accordance with the Cochrane Handbook for Systematic Reviews (Higgins et al. 2024) and reported in line with PRISMA guidelines (Page et al. 2021) to identify studies reporting the prevalence and characteristics of BPD in adults with RCC or ILO. The review protocol was registered with the international Prospective Register of Systematic Reviews (PROSPERO) registration number: CRD420261419679.

### 2.1 Search Strategy

To identify relevant studies, the following databases were searched: MEDLINE (via EBSCO), the Cumulative Index to Nursing and Allied Health Literature (CINAHL) (via EBSCO), PsycINFO (via EBSCO), the Cochrane Central Register of Controlled Trials (CENTRAL), and Web of Science.

Search strategies combined controlled vocabulary (e.g. subject headings in MEDLINE and CINAHL) and free‐text terms relating to (1) breathing pattern disorder (including terms such as “breathing pattern disorder”, “dysfunctional breathing”, “disordered breathing”, and “hyperventilation”), and (2) upper airway conditions (including “chronic cough”, “refractory cough”, “inducible laryngeal obstruction”, “vocal cord dysfunction”, and related synonyms such as VCD (vocal cord dysfunction) and PVFM (paradoxical vocal fold movement)). Search strategies were adapted for each database to reflect differences in indexing and functionality; for example, subject headings were used where available (MEDLINE, CINAHL), while text word searches were prioritised in PsycINFO due to differences in indexing. Simplified keyword strategies were used for CENTRAL and Web of Science. The full search strategies and results are shown in Table A1.

### 2.2 Eligibility Criteria

No outcome‐specific search terms (e.g. prevalence or incidence) were included due to anticipated variability in reporting; instead, studies reporting any data on the prevalence or characteristics of BPD were identified and relevant data was extracted during screening.

Studies were included if they:

- Included adults aged ≥18 years. An exception was applied to otherwise eligible studies of Exercise‐Induced Laryngeal Obstruction (EILO), irrespective of participant age, because EILO is predominantly investigated in younger populations. These studies were retained as a distinct subgroup and synthesised separately from the adult RCC and ILO evidence.
- Included participants with physician‐diagnosed RCC or ILO, with laryngoscopy required for ILO diagnosis.
- Accepted any terminology describing abnormal breathing patterns, provided the breathing changes were clearly described.
- Were conducted in any country or healthcare setting.
- Published in English in the last ten years to ensure relevance to contemporary clinical practice and diagnostic approaches.
- Were published in peer‐reviewed journals.

Studies were excluded if they:

- Included participants aged under 18 years, unless the study investigated EILO and otherwise met the eligibility criteria.
- Did not report prevalence and/or characteristic data relating to BPD within an RCC or ILO population.
- Primarily attributed breathing abnormalities to other conditions, including lower airway diseases such as asthma, COPD, or COVID‐19.
- Were single case studies or conference abstracts.

The reference lists of included studies were screened for additional potentially relevant records. This process identified several related conference abstracts, but no additional full‐text peer‐reviewed studies meeting the eligibility criteria. Other grey literature was not systematically searched.

### 2.3 Study Selection

All records were imported into Rayyan, a web‐based systematic review management platform, for duplicate removal and screening, which was completed by the primary reviewer. All titles and abstracts were then screened against predefined inclusion and exclusion criteria. To enhance screening reliability, a randomly generated 10% sample of records, created using Rayyan's sampling function, was independently screened by two additional reviewers. Reviewer agreement was 84% (21/25 records). The four disagreements related to interpretation of the eligibility criteria and were resolved through discussion and consensus agreement.

Figure 1 details the study selection using the Preferred Reporting Items for Systematic Reviews and Meta‐analyses (PRISMA) flow diagram (Page et al. 2021).

**FIGURE 1:**
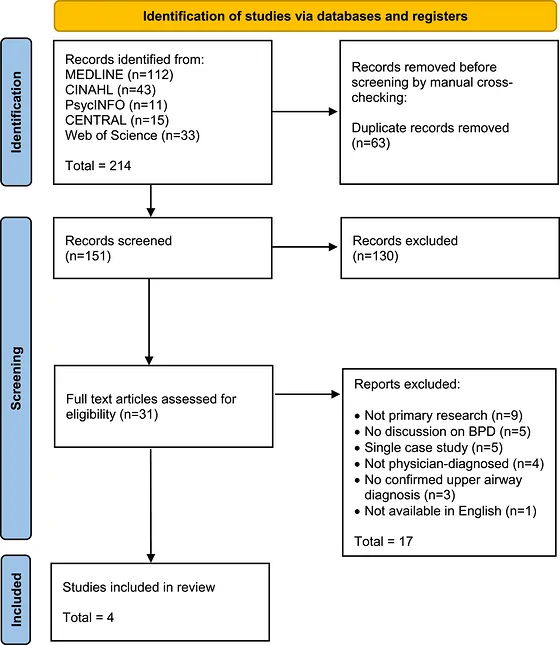
PRISMA Flow Diagram of search process. CINAHL, The Cumulative Index to Nursing and Allied Health Literature; CENTRAL, Cochrane Central Register of Controlled Trials; BPD, Breathing pattern disorder; PRISMA, Preferred Reporting Items for Systematic Reviews and Meta‐Analyses.

### 2.4 Data Extraction

Once the eligible studies were selected, a data extraction form was created in Microsoft Excel to gather the following information:

1. General information: type of upper airway disorder, authors, publication year.
2. Information on participants and setting.
3. Information on how the upper airway disorder was diagnosed.
4. Information on how the assessment of breathing pattern features was completed.
5. Prevalence information of BPD within upper airway disorder.
6. Characteristics of BPD described.

### 2.5 Data Synthesis

Due to heterogeneity in study populations, BPD definitions, and assessment methods, statistical pooling was not considered appropriate. Findings were therefore synthesised narratively. Results were organised according to clinical population (RCC, ILO, and EILO) and summarised with reference to prevalence estimates and reported BPD characteristics.

### 2.6 Quality Appraisal

Methodological quality of included studies was assessed using the Joanna Briggs Institute Critical Appraisal Checklist for Studies Reporting Prevalence Data (Munn et al. 2020), with results presented in Table A2. The certainty of evidence was evaluated using the GRADE approach (Goldkuhle et al. 2025), with results presented in Table A3. These assessments were used to inform interpretation of findings and were not used as criteria for study exclusion.

## 3 Results

The results were collated, summarised, and reported according to whether they related to RCC, ILO, or EILO, as shown in Table 1. Although 4 unique articles met the inclusion criteria, 2 studies reported data across more than one diagnostic group, resulting in 6 analysable cohorts.

**TABLE 1:** Results.

Type of upper airway disorder	Author, publication year, location	Information on participants and setting	Information on how upper airway disorder was diagnosed	Information on how assessment of breathing pattern features was completed	Prevalence information of BPD within upper airway disorder	Characteristics of BPD described
Refractory Chronic Cough	Jeffery et al. 2024 (Australia)	542 participants referred to a tertiary speech pathology department for behavioural treatment of Chronic Refractory Cough (CRC). Mean age 58 years old (± 16). 77% female (*n =* 418/542), male 23% (*n =* 124/542).	CRC diagnosed by respiratory physician and defined as a cough persisting for >8 weeks and refractory to medical management.	Clinical assessment by Speech Pathologist of breathing completed while sitting. Breathing region (thoracic vs abdominal), route (oral vs nasal), and rate were used to describe breathing pattern features. Additional features that were noted included stridor or noisy breathing, breath holding, and poor coordination of respiration and phonation.	73% (*n =* 395/542) patients had chronic refractory cough and at least one breathing abnormality (region, route, or rate).	Abnormalities found: 69% (*n =* 372/542) region, 33% (*n =* 178/542) route, 24% (*n =* 128/542) rate. 29% (*n =* 159/542) had region and route abnormalities. 23% (*n =* 123/542) had region and rate abnormalities. 8% (*n =* 43/542) had route and rate abnormalities. 8% (*n =* 42/542) had all three aspects abnormal. It was noted that 16% (*n =* 87/542) also had paradoxical vocal fold movement. Researchers found an association between abnormal breathing pattern features and laryngeal motor function, demonstrated by reduced maximum phonation time values.
Refractory Chronic Cough	Vertigan et al. 2020 (Australia)	39 participants referred to a speech pathology department for treatment of chronic cough. Mean age 58 years old (± 14). 79% female (*n =* 31/39), male 21% (*n =* 8/39).	Clinician diagnosis of chronic cough. Cough persisting for longer than eight weeks that was refractory to guideline based medical management.	Assessment of breathing completed while sitting by Speech Pathologist. Assessment of tidal respiration and speech of breathing pattern, route of breathing, region of most movement (upper chest/clavicular or abdominal movement), breath support and laryngeal muscle tension if present.	No overall prevalence provided. 39 participants in study.	Abnormalities found: 33% (*n =* 13/39) oral breathing, 21% (*n =* 8/39) clavicular breathing, 18% (*n =* 7/39) breath holding, 18% (*n =* 7/39) stridor, 15% (*n =* 6/39) reduced breath support, 8% (*n =* 3/39) effortful inhalation, and 8% (*n =* 3/39) shallow breathing, Strong correlation reported between number of BPD abnormalities and cough frequency. No significant correlation between the number of breathing pattern abnormalities and maximum phonation time. 69% (*n =* 27/39) had moderate‐severe paradoxical vocal fold movement present during laryngoscopy. Inspiration was affected in 30% (*n =* 8/27) and expiration in 52% (*n =* 14/27). Both phases of breathing were affected in 19% (*n =* 5/27) but within different breathing cycles.
ILO	Jeffery et al. 2024 (Australia)	92 participants referred to a tertiary speech pathology department for behavioural treatment of ILO. Mean age 53 years old (± 18). 75% female (*n =* 69/92), male 25% (*n =* 23/92).	ILO diagnosed by laryngoscopy, as well as clinical features such as inspiratory dyspnoea, noisy breathing, and throat tightness.	Clinical assessment by Speech Pathologist of breathing completed while sitting. Breathing region (thoracic vs abdominal), route (oral vs nasal), and rate were used to describe breathing pattern features. Additional features that were noted included stridor or noisy breathing, breath holding, and poor coordination of respiration and phonation.	69% (*n =* 63/92) ILO and at least one breathing abnormality (either region, route, or rate).	Abnormalities found: 66% (*n =* 60/92) region, 26% (*n =* 24/92) route, 24% (*n =* 22/92) rate. 24% (*n =* 22/92) had region and route abnormalities. 24% (*n =* 22/92) had region and rate abnormalities. 2% (*n =* 2/92) had route and rate abnormalities. 2% (*n =* 2/92) had all three aspects abnormal. Found an association between abnormal breathing pattern features and laryngeal motor function, demonstrated by reduced maximum phonation time values.
ILO	Vertigan et al. 2020 (Australia)	51 participants referred to speech pathology a department for VCD. Mean age 57 years old (± 17). 80% female (*n =* 41/51), male 20% (*n =* 10/51).	Confirmed signs of moderate‐severe paradoxical vocal fold movement (PVFM) on functional transnasal laryngoscopy, a finding conceptually consistent with ILO although the term ILO was not used in the original study.	Assessment of breathing completed while sitting by Speech Pathologist. Assessment of tidal respiration and speech of breathing pattern, route of breathing, region of most movement (upper chest/clavicular or abdominal movement), breath support and laryngeal muscle tension if present.	89% (*n =* 46/51) had at least one breathing pattern abnormality. 42% (*n =* 22/51) had three or more abnormalities.	Abnormalities found: 33% (*n =* 17/51) oral breathing, 18% (*n =* 9/51) clavicular breathing, 18% (*n =* 9/51) stridor, 16% (*n =* 8/51) shallow breathing, 16% (*n =* 8/51) reduced breath support, 12% (*n =* 6/51) effortful inhalation, and 12% (*n =* 6/51) breath holding. Number of breathing pattern changes were correlated with voice impact measures including CAPE‐V (Consensus Auditory‐Perceptual Evaluation of Voice) (*r* = 0.38, *p* = 0.006) and maximum phonation time (*r* = 0.32, *p* = 0.025). Laryngeal muscle tension was present in 39% (*n =* 20/51) of participants.
EILO	Lie et al. 2021 (Norway)	24 participants with moderate‐severe EILO randomly selected from a specialist EILO register at a university hospital. Mean age 17 years (± 6.1), 92% female (*n =* 22/24), 8% male (*n =* 2/24). Compared with 20 highly active individuals without respiratory complaints. Mean age 24 years (± 6.4), 55% female (*n =* 11/20), 45% male (*n =* 9/20).	Laryngoscopy treadmill exercise tests, including ergospirometry.	Analyses of breathing patterns from an ergospirometry tests using mathematical modelling	Few clear‐cut differences reported between EILO and non‐EILO groups. The non‐EILO group had no exercise‐related respiratory complaints and did not have visual signs of laryngeal obstruction on laryngoscopy.	EILO group generally had a prolonged inspiratory time, lower inspiratory flow rate, and lower breathing frequency. Also, slightly increased expiratory time which might be explained by lower breathing frequency. Tidal volume at peak exercise was similar across both groups. Hyperventilation did not appear to be a driving factor in EILO.
EILO	Milstein et al. 2023 (USA)	77 athletes referred to a specialist voice centre for suspected EILO. Mean age 13 years (± 3.36), 73% female (*n =* 56/77), 25% male (*n =* 19/77), 3% non‐binary (*n =* 2/77). Compared with 58 athletes without respiratory complaints. Mean age 18 years (± 1.60), 67% female (*n =* 39/58), 33% male (*n =* 19/58).	EILO confirmed on videoendoscopy. Test undertaken at rest, during performance of high ventilatory output tasks of heavy breathing/panting, and after strenuous exercise.	Milstein‐Breathing Pattern Assessment Index (M‐BPAI) score of >8 completed by Speech Pathologists from video recordings.	77% (*n =* 41/53) participants with EILO confirmed via videoendoscopy exhibited BPD on provocation. However, also 79% (*n =* 19/24) participants who did not have a diagnosis of EILO on videoendoscopy, but referred with EILO symptoms, also demonstrated BPD under provocation.	Little difference noted between the two groups. During increased ventilation individuals typically switched from nasal to oral breathing. EILO group had more shoulder displacement, head tilt, strap muscle contraction, and audible inhale. No significant difference in abdominal movement.

Abbreviations: ±, Standard Deviation; BPD, Breathing pattern disorder; CRC, Chronic Refractory Cough; EILO, Exercise‐induced Laryngeal Obstruction; ILO, Inducible Laryngeal Obstruction.

In total, 2 articles discussed RCC, 2 discussed ILO, and 2 were related to EILO. EILO is a subtype of ILO in which laryngeal narrowing occurs specifically during exercise and, in the included studies, was confirmed using exercise‐provoked laryngoscopic assessment. Participants underwent exercise testing, typically involving treadmill exercise to maximal exertion, with EILO identified by the presence of glottic and/or supraglottic obstruction during exercise. Unlike ILO, which may be triggered by a broader range of factors (e.g. irritants, environmental factors, or emotional triggers), EILO is specifically associated with exercise as the provoking factor. Furthermore, EILO studies frequently involve adolescent populations, and participants in the included EILO studies fell outside the adult‐only eligibility criterion applied to the primary review population. Consequently, findings from the EILO studies were analysed separately from the RCC and ILO evidence.

### 3.1 Findings by Clinical Population

#### 3.1.1 Refractory Chronic Cough (RCC)

As summarised in Table 1, two articles were found relating to RCC. Of these, Jeffery et al. (2024) reported that 73% (*n =* 395/542) of patients with CRC had at least one breathing abnormality. CRC was considered conceptually equivalent to RCC. No overall prevalence estimate of BPD in their RCC cohort was reported by Vertigan et al. (2020). Between the two studies, there was variation in whether breathing region (thoracic vs abdominal) or breathing route (oral vs nasal) was the most common abnormality; however, both studies identified these as prominent features of altered breathing patterns.

Vertigan et al. (2020) reported that 69% (*n =* 27/39) of participants with RCC demonstrated moderate‐severe paradoxical vocal fold movement during laryngoscopy, a finding conceptually consistent with ILO. In contrast, Jeffery et al. (2024) reported a lower prevalence of 16% (*n =* 87/542). Vertigan et al. (2020) also reported a correlation between BPD abnormalities and cough frequency.

Jeffery et al. (2024) identified an association between abnormal breathing pattern features and reduced maximum phonation time (MPT), whereas Vertigan et al. (2020) assessed this relationship but did not identify a significant association.

#### 3.1.2 Inducible Laryngeal Obstruction (ILO)

Two articles were found discussing ILO, Jeffery et al. (2024) reported that 69% (*n =* 63/92) of patients with ILO had at least one breathing abnormality, while Vertigan et al. (2020) reported a higher prevalence of 89% (*n =* 46/51) in participants with vocal cord dysfunction (VCD), a condition conceptually overlapping with ILO. Similar to the RCC studies, there was variation between studies regarding whether breathing region or breathing route abnormalities were more common; however, again both studies identified these as prominent features of altered breathing patterns.

Both identified associations between abnormal breathing pattern features and reduced MPT. Vertigan et al. (2020) also reported that 39% (*n =* 20/51) of participants demonstrated laryngeal muscle tension, a feature not measured by Jeffery et al. (2024).

#### 3.1.3 Exercise‐Induced Laryngeal Obstruction (EILO)

Two articles reported findings relating to EILO and were analysed separately from the adult RCC and ILO evidence. Both included younger populations, participants in Lie et al. (2021) had a mean age of 17 years, while those referred with suspected EILO in Milstein et al. (2023) had a mean age of 13 years. The findings should therefore not be interpreted as evidence specifically relating to adults.

In the included studies, EILO was identified using laryngoscopic visualisation. Exercise protocols varied between studies and included continuous laryngoscopy during treadmill exercise with ergospirometry (Lie et al. 2021) and videoendoscopic assessment performed at rest, during high ventilatory output tasks (heavy breathing/panting), and following strenuous exercise (Milstein et al. 2023).

Fewer differences were reported between EILO and comparison groups. Lie et al. (2021) included a control group of healthy individuals with no respiratory complaints, whereas Milstein et al. (2023) included participants with suspected EILO but no confirmed diagnosis on videoendoscopy. Both studies reported minimal differences in breathing characteristics between these groups.

## 4 Discussion

A limited number of studies were identified in this search, which reflects the relatively small body of published research explicitly examining BPD within RCC and ILO populations. It is possible that, rather than reflecting a newly recognised phenomenon, breathing dysfunction has historically been recognised clinically but not consistently conceptualised or reported as BPD within clinical practice or the published literature. This is supported by the fact that BPD remains inconsistently defined across the literature, and there is currently no universally accepted diagnostic framework (Grillo et al. 2025), which may also contribute to the limited number of studies on this topic. In addition, reference standard diagnosis of ILO and EILO requires specialist assessment techniques, including laryngoscopy and provocation testing (Halvorsen et al. 2017), which may not be routinely available outside specialist centres (Haines et al. 2020). This likely restricts access to appropriately characterised patient populations and contributes to small sample sizes observed across published studies.

An important consideration when interpreting these findings is that BPD assessments were undertaken under different conditions. The RCC and ILO studies predominantly assessed breathing patterns at rest, whereas the EILO studies assessed breathing patterns during exercise or exercise‐related provocation. For example, Milstein et al. (2023) used the Milstein Breathing Pattern Assessment Index (M‐BPAI), an observational tool developed to identify breathing pattern abnormalities during high‐ventilatory tasks in athletes with suspected EILO. The differing assessment contexts and methodologies may have influenced how breathing pattern abnormalities were identified, as these abnormalities may present differently at rest, during exercise, or during other forms of provocation, potentially contributing to variation in findings between studies.

Despite these differences, the included studies suggest that BPD is commonly reported in adults with RCC and ILO. Findings from the separately analysed, predominantly younger EILO populations were less clearly differentiated from their comparison groups. A consideration when interpreting these findings is that the assessments of BPD in most of the included studies were predominantly conducted by Speech and Language Pathologists/Therapists. As a result, reported prevalence and characteristic data may reflect the clinical focus of SLT‐led assessments. Clinicians trained in assessment of laryngeal function and breathing‐phonation coordination may be more likely to identify subtle abnormalities (Haines et al. 2021), which may influence prevalence estimates, as identification of BPD is known to be inconsistent and dependent on assessment approach (Bondarenko et al. 2026). This may also indicate that BPD is under‐recognised in settings without access to specialist SLT or physiotherapy assessment, with variability in assessment contributing to inconsistent identification and potential underestimation of prevalence (Bondarenko et al. 2026). This supports the need for assessment tools that are both clinically sensitive and applicable across disciplines. Future research would benefit from standardised descriptions of breathing region, route, and rate; clearly defined assessment conditions; and evaluation of inter‐rater reliability across professional groups.

Across the included studies, abnormalities in breathing region (thoracic vs abdominal) and breathing route (oral vs nasal) were most frequently reported. The recurrence of these specific features across studies suggests some consistency in the breathing abnormalities reported across RCC and ILO populations. A schematic summary of the BPD characteristics reported across the included studies is shown in Figure 2.

**FIGURE 2:**
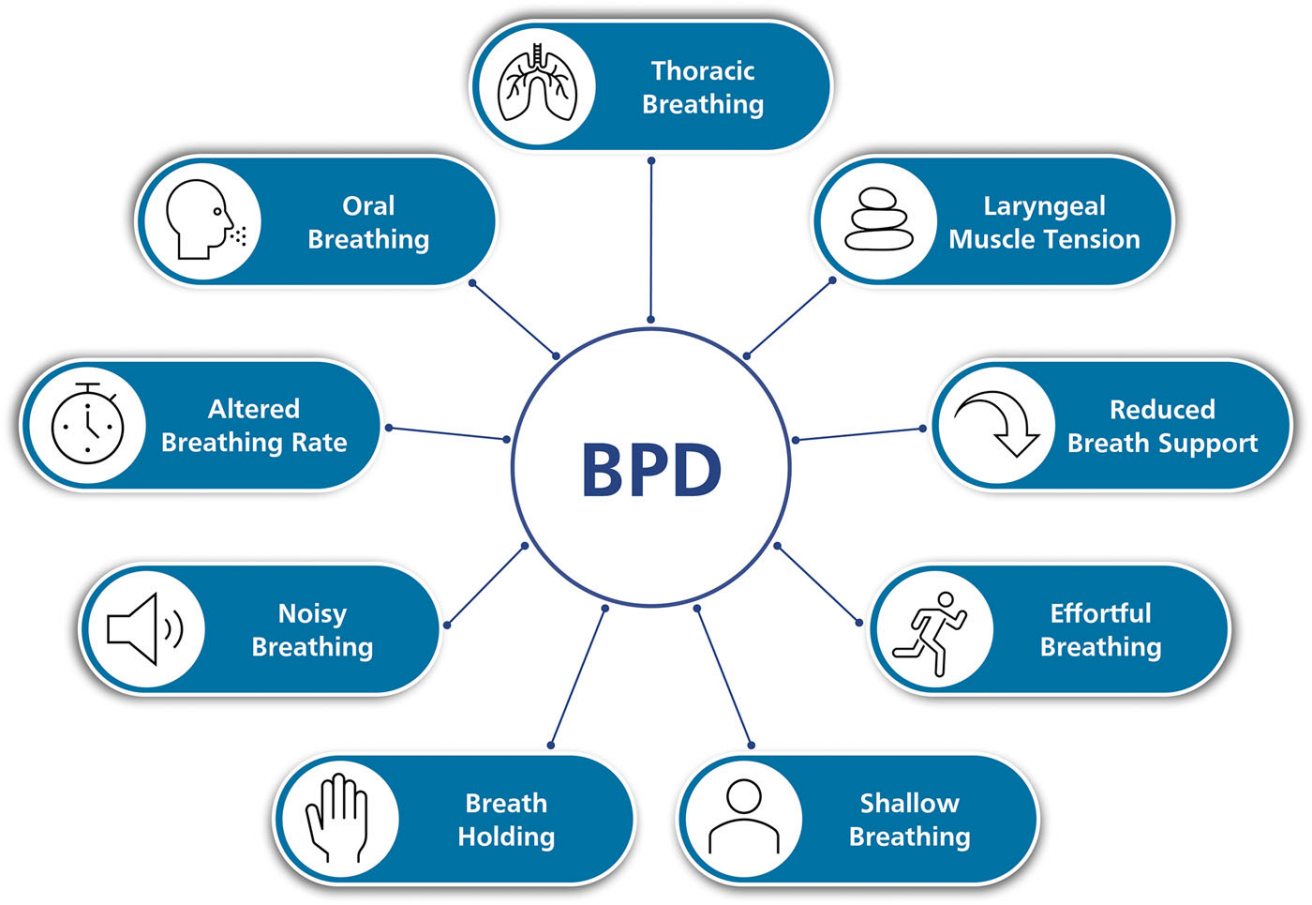
Breathing pattern disorder (BPD) characteristics.

The included studies suggest associations between BPD, laryngeal dysfunction, and respiratory symptoms in RCC and ILO populations. The presence of paradoxical vocal fold movement within RCC cohorts, a hallmark feature of ILO, suggests overlap in symptom presentation between these conditions. Additionally, a relationship between BPD and cough frequency was also observed, with cough also recognised as a typical clinical feature in ILO populations (Halvorsen et al. 2017). This is consistent with proposals that chronic cough and paradoxical vocal fold movement may represent different manifestations of a shared underlying condition (Vertigan et al. 2006).

Furthermore, associations between BPD and maximum phonation time in some studies indicate that these relationships may extend beyond cough and airway obstruction to include voice function, which also depends on coordinated laryngeal and respiratory activity. In the literature, laryngeal dysfunction is recognised to encompass voice, cough, and ILO (Hull et al. 2016). Together, these findings suggest an association between dysfunctional breathing and laryngeal function, with the possibility of a bidirectional relationship whereby BPD may contribute to, and result from, laryngeal dysfunction. However, current evidence reflects association rather than causation.

In the literature, there have been several suggestions proposed to explain this. Selby et al. (2019) propose that abnormal breathing patterns can contribute to the sensation of tightness and dyspnoea, which may mimic symptoms of ILO. Denton et al. (2019) discuss that BPD may stem from maladaptive responses involving suboptimal postures or learned techniques developed as a coping mechanism. In these cases, the breathing patterns might be used to compensate for breathlessness or an increased perception of airflow resistance, which over time could become habitual (Jeffery et al. 2024).

A range of clinical and physiological factors have been reported within the literature as contributing to the presentation of both BPD and upper airway conditions. There are a broad range of factors which impact on BPD and ILO, including lower airway disease (Thomas et al. 2005), nasal disease (Kenn and Balkissoon 2011), reflux (Spyridoulias et al. 2015), psychological factors (Leo and Konakanchi 1999), organic causes, cardiac, and neurological conditions (Ludlow et al. 2023). Factors influencing chronic cough caseloads include, smoking, Angiotensin‐Converting Enzyme Inhibitors (ACEI) medications, obesity (Kim et al. 2025), Obstructive Sleep Apnoea (Satia et al. 2025), chronic aspiration, infection, and irritant exposure (Parker and Butler 2025). These factors are likely to interact across breathing and laryngeal functions, contributing to the complexity of symptom presentation and supporting a multifactorial conceptualisation of how BPD and laryngeal dysfunction sit within a wider network of interacting physiological and psychosocial influences.

Figure 3 presents a conceptual framework illustrating possible associations between dysfunctional breathing, laryngeal function, and contributing factors discussed within the wider literature. These factors are likely to interact with breathing and laryngeal dysfunction in complex ways, potentially contributing to the maintenance or exacerbation of symptoms; however, the current evidence does not allow conclusions regarding the direction or causality of these relationships. The figure is intended to illustrate potential relationships rather than causal pathways.

**FIGURE 3:**
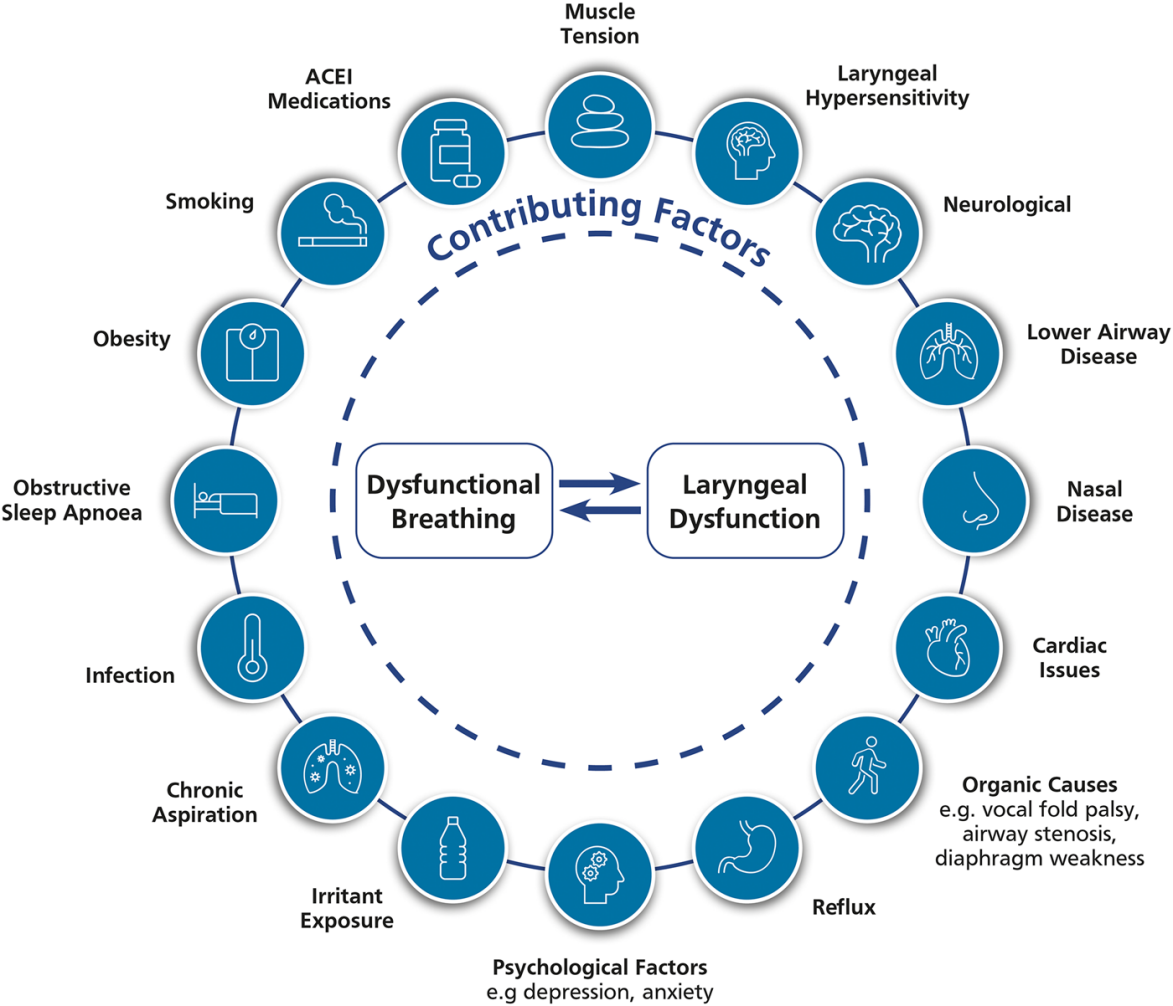
Conceptual framework illustrating potential associations between dysfunctional breathing, laryngeal function, and contributing factors reported in the literature. ACEI, Angiotensin‐Converting Enzyme Inhibitors.

In contrast, findings in EILO are less consistent and suggest that BPD may not be specific to this condition, as similar breathing pattern changes are also observed in comparison groups without confirmed EILO, indicating that these changes may reflect a more general response to respiratory challenge or exertion, rather than being a condition‐specific feature. These studies also suggest that hyperventilation may be less prominent in EILO, further reinforcing findings from the RCC and ILO studies that breathing rate appears to be a less prominent feature across breathing pattern changes.

### 4.1 Strengths and Limitations

This review provides the first comprehensive synthesis of prevalence data on BPD within upper airway disorder populations. A key strength lies in the systematic search strategy across five electronic databases, with independent screening by three reviewers. However, the exclusion of conference abstracts, single case‐studies, and non‐English publications may have limited the breadth of available evidence. Application of the Joanna Briggs Institute Critical Appraisal Checklist for Studies Reporting Prevalence Data (Munn et al. 2020) indicated generally acceptable methodological quality across included studies; however, GRADE assessment (Goldkuhle et al. 2025) indicated low to very low certainty of evidence owing to methodological heterogeneity, indirectness, imprecision, and the small number of studies available.

An additional limitation is that all included studies were conducted within specialist referral services. Consequently, reported prevalence estimates may be influenced by referral bias, as patients attending specialist upper airway clinics are likely to differ from broader RCC or ILO populations. The prevalence reported within this review should therefore not be interpreted as representative of community or population‐level prevalence. Furthermore, some included studies were produced by overlapping research teams, which may have contributed to similarities in assessment approaches across studies.

Heterogeneity was influenced by variation in the definitions of BPD, diagnostic approaches, and assessment methods used across studies, which may have affected the comparability of prevalence estimates and reported BPD characteristics. In addition, most included participants were female, which may limit the generalisability of findings to male populations. Furthermore, two EILO studies involving younger populations were retained under an exception to the age criterion and analysed separately from the adult RCC and ILO evidence. Their findings should therefore not be interpreted as representative of adult EILO populations.

A further limitation is that PROSPERO registration was completed after the review had been commenced; however, methods were predefined and reported transparently within the registration. Any methodological refinements made during the review process are reported in the final manuscript. Despite these limitations, the synthesis offers a valuable overview of this emerging area and provides a foundation for future research.

## 5 Conclusion

BPD appears to be a common comorbidity in adults with RCC and ILO, highlighting the importance of incorporating breathing pattern assessment into routine clinical management. Overlap in presentation between BPD, RCC, and ILO may complicate assessment and treatment, particularly in settings without access to laryngoscopy. Reliance on patient‐reported symptoms alone may increase the risk of diagnostic crossover, reinforcing the need for clinician awareness of these interrelated conditions. The development of standardised diagnostic protocols to support differential diagnosis should be a priority for future research.

Understanding the relationship between dysfunctional breathing patterns and laryngeal dysfunction is important, as misattribution of BPD symptoms to structural airway disorders may lead to unnecessary investigations or ineffective management. A holistic approach to breathing and laryngeal dysfunction is beneficial, with recognition that individuals may present with different patterns of breathing dysfunction, supporting the need for a personalised therapeutic approach. By identifying and addressing abnormal breathing patterns, clinicians may be better able to support the restoration of optimal laryngeal function in patients, a core aspect of upper airway management. Further research exploring whether BPD acts as a cause, consequence, or maintaining factor in upper airway dysfunction would be valuable.

Overall, this review addresses a gap in the literature regarding the prevalence and characteristics of BPD within upper airway populations. BPD appears to be a frequent comorbidity in RCC and ILO, with overlapping clinical features observed across conditions. These findings emphasise the need for standardised, provocation‐sensitive assessment protocols to support accurate clinical differentiation, particularly in centres without access to laryngoscopy. Clinicians should be aware of the potential for crossover between BPD, RCC, and ILO and consider incorporating comprehensive breathing assessment into routine upper airway evaluation. SLTs are well positioned to support the development of clinically feasible, standardised BPD assessment pathways within multidisciplinary upper airway services.

## Funding

Kirsty Bui (Predoctoral Award Fellow, NIHR501164) and Gemma Clunie (Senior Clinical and Practitioner Research Award Fellow, NIHR304447) were funded by the National Institute for Health and Care Research (NIHR) for this research project. Gemma Clunie also acknowledges support of the NIHR Imperial Biomedical Research Centre (BRC). The views expressed in this publication are those of the authors and not necessarily those of the NIHR, NHS, or the UK Department of Health and Social Care.

## Conflicts of Interest

The authors declare no conflicts of interest.

## Supporting information




**Supporting Information**:jlcd70365‐sup‐0001‐SuppMat.docx

## Data Availability

All data relevant to the study are included in the article or uploaded as Supporting Information.

## 1

A1, A2, A3

**TABLE A1:** Search strategy and results.

MEDLINE		
Search	Terms	Results
S8	S3 AND S6 (10 year limit)	112
S7	S3 AND S6	235
S6	S4 OR S5	30 338
S5	XB ((cough* N3 (chronic OR persistent OR refractory OR unexplained))) OR (“induc* laryngeal obstruction” OR ILO OR VCD OR PVFM OR PVCM OR “vocal cord dysfunction”)	14 019
S4	(MH “Cough” OR MH “Chronic Cough”) OR MH “Vocal Cord Dysfunction”	20 289
S3	S1 OR S2	29 289
S2	XB (breath* N3 (pattern* OR disorder* OR dysfunction* OR abnormal*) OR (dysfunctional breath* OR disordered breath* OR hyperventilat*)	28 572
S1	MM “Hyperventilation”	3293

CINAHL		
Search	Terms	Results
S8	S3 AND S6 (10 year limit)	43
S7	S3 AND S6	69
S6	S4 OR S5	10 478
S5	XB ((cough* N3 (chronic OR persistent OR refractory OR unexplained))) OR (“induc* laryngeal obstruction” OR ILO OR VCD OR PVFM OR PVCM OR “vocal cord dysfunction”)	3496
S4	MH “Cough” OR MH “Chronic Cough”) OR MH “Vocal Cord Dysfunction”	8365
S3	S1 OR S2	6824
S2	XB (breath* N3 (pattern* OR disorder* OR dysfunction* OR abnormal*)) OR abnormal*OR dysfunctional breath* OR disordered breath* OR hyperventilat*)	6433
S1	MH “Hyperventilation”	934

APA PsycINFO		
Search	Terms	Results
S4	S1 AND S2 (10 year limit)	11
S3	S1 AND S2	20
S2	XB (cough* OR “chronic cough”) OR (“induc* laryngeal obstruction” OR ILO OR VCD OR PVFM OR PVCM OR “vocal cord dysfunction”)	2379
S1	XB (“breathing pattern*” OR “breathing pattern disorder*” OR “breathing pattern dysfunction*” OR “dysfunctional breathing” OR “disordered breathing”) OR (breath* N3 (pattern* OR dysfunction* OR disorder* OR abnormal*)) OR (hyperventilat* N3 (breath* OR syndrome)))	2708

Cochrane Central Register		
Search	Terms	Results
#9	#4 AND #7 with Cochrane Library publication date from Jan 2016 to Apr 2026	15
#8	#4 AND #7	20
#7	#5 OR #6	4210
#6	(“breathing pattern” OR “dysfunctional breathing” OR “disordered breathing” OR hyperventilation)	4201
#5	MeSH descriptor: [Hyperventilation] explode all trees	335
#4	#1 OR #2 OR #3	3228
#3	(“chronic cough” OR “vocal cord dysfunction” OR “inducible laryngeal obstruction” OR ILO OR VCD OR PVFM)	1620
#2	MeSH descriptor: [Cough] explode all trees	1910
#1	MeSH descriptor: [Chronic Cough] explode all trees	43

Web of Science		
Search	Terms	Results
#2	TS = ((“breathing pattern*” OR “dysfunctional breathing” OR “disordered breathing” OR hyperventilation) AND (“chronic cough” OR “refractory cough” OR “inducible laryngeal obstruction” OR ILO OR “vocal cord dysfunction” OR VCD OR PVFM)) Refined By:Final Publication Year: 2026 or 2025 or 2024 or 2023 or 2021 or 2018 or 2020 or 2019 or 2022 or 2017 or 2016	33
#1	TS = ((“breathing pattern*” OR “dysfunctional breathing” OR “disordered breathing” OR hyperventilation) AND (“chronic cough” OR “refractory cough” OR “inducible laryngeal obstruction” OR ILO OR “vocal cord dysfunction” OR VCD OR PVFM))	68

**TABLE A2:** JBI critical appraisal tools for use in JBI systematic reviews checklist for prevalence studies (Munn et al. 2020).

Quality assessment indicators	Selected studies			
	Jeffery et al. (2024)	Vertigan et al. (2020)	Lie et al. (2021)	Milstein et al. (2023)
Q1. Was the sample frame appropriate to address the target population?	Y	Y	Y	Y
Q2. Were study participants sampled in an appropriate way?	Y	U	Y	Y
Q3. Was the sample size adequate?	Y	U	U	U
Q4. Were the study subjects and the setting described in detail?	Y	Y	Y	Y
Q5. Was the data analysis conducted with sufficient coverage of the identified sample?	Y	Y	Y	Y
Q6. Were valid methods used for the identification of the condition?	Y	Y	Y	U
Q7. Was the condition measured in a standard, reliable way for all participants?	U	U	Y	U
Q8. Was there appropriate statistical analysis?	Y	Y	Y	Y
Q9. Was the response rate adequate, and if not, was the low response rate managed appropriately?	Y	U	U	Y
Overall appraisal	Include	Include	Include	Include

*Note*: Lie et al. (2021) primarily examined breathing‐pattern characteristics and did not estimate the prevalence of BPD. The JBI checklist was applied to assess methodological quality only.

Abbreviations: *N =* No; U = unclear; Y = yes.

**TABLE A3:** GRADE assessment of certainty of evidence Goldkuhle et al. (2025).

Outcome		Studies	Certainty (GRADE)	Reason for rating
1	Prevalence of BPD in RCC	2	Low	Observational evidence; potential referral bias due to specialist‐service recruitment; limited number of studies.
2	BPD characteristics in RCC	2	Very low	Observational evidence, variation in assessment methods and reporting, limited number of studies.
3	Prevalence of BPD in ILO	2	Low	Observational studies, small sample sizes, specialist‐centre recruitment.
4	BPD characteristics in ILO	2	Very low	Observational evidence, heterogeneity in assessment methods and outcome reporting, small evidence base.
5	Prevalence of BPD in EILO	1	Very low	Single observational study, indirectness due to younger population, small sample size.
6	BPD characteristics in EILO	2	Very low	Small observational evidence base, indirectness due to younger populations, differing assessment approaches and comparison groups.
